# Unexpected Remission of Bilateral Hyperaldosteronism After Unilateral Cortisol-Producing Adenoma Resection: A Report of Two Cases

**DOI:** 10.1155/crie/9941688

**Published:** 2025-06-18

**Authors:** Kazunari Hara, Masanori Murakami, Kumiko Shiba, Kazutaka Tsujimoto, Chikara Komiya, Kenji Ikeda, Kurara Yamamoto, Towako Taguchi, Takumi Akashi, Soichiro Yoshida, Kenichi Ohashi, Yasuhisa Fujii, Tetsuya Yamada

**Affiliations:** ^1^Department of Molecular Endocrinology and Metabolism, Graduate School of Medical and Dental Sciences, Institute of Science Tokyo, Tokyo 113-8510, Japan; ^2^The Center for Personalized Medicine for Healthy Aging, Institute of Science Tokyo, Tokyo, Japan; ^3^Department of Human Pathology, Graduate School of Medical and Dental Sciences, Institute of Science Tokyo, Tokyo, Japan; ^4^Department of Comprehensive Pathology, Graduate School of Medical and Dental Sciences, Institute of Science Tokyo, Tokyo, Japan; ^5^Department of Diagnostic Pathology, Graduate School of Medical and Dental Sciences, Tokyo Medical and Dental University, Tokyo, Japan; ^6^Division of Surgical Pathology, Tokyo Medical and Dental University Hospital, Tokyo, Japan; ^7^Department of Urology, Graduate School of Medical and Dental Sciences, Institute of Science Tokyo, Tokyo, Japan

**Keywords:** adrenal tumor, Cushing's syndrome, mild autonomous cortisol secretion, primary aldosteronism

## Abstract

Primary aldosteronism (PA) is the most frequent cause of secondary hypertension. PA is primarily categorized into two subtypes: The unilateral subtype, which mainly consists of aldosterone-producing adenoma (APA) and the bilateral subtype, in which aldosterone is overproduced by both adrenal glands. Rarely, does the bilateral form of PA coexists with a cortisol-producing adenoma (CPA), as documented by previous reports. In this context, we present two cases wherein the preoperative diagnosis identified the bilateral form of PA accompanied by a unilateral CPA. However, postresection of the CPA, unexpected resolution of the bilateral form of PA was observed in both patients. Case 1：A 57-year-old female presented with overt Cushing's syndrome attributed to a left adrenal tumor and concomitant bilateral PA. Laparoscopic left adrenalectomy was performed for the treatment of Cushing's syndrome. Case 2：A 67-year-old female diagnosed with a left adrenal tumor with coexisting bilateral PA. The left adrenal tumor exhibited mild autonomous cortisol secretion (MACS) and given the increase in tumor size, laparoscopic left adrenalectomy was undertaken. After 1 year of surgery, we conducted a captopril challenge test (CCT) on both patients, revealing that neither satisfied the diagnostic criteria for PA. In patients where unilateral CPA coexisted with bilateral PA, unilateral adrenalectomy may provide remission of not only the autonomous cortisol secretion but also bilateral PA. Consequently, postoperative evaluation of PA assumes significance.

## 1. Introduction

Primary aldosteronism (PA) is the most frequent cause of secondary hypertension. An aldosterone-producing adenoma (APA) represents the unilateral disease, while bilateral forms, in which aldosterone is overproduced by both adrenal glands, constitute common subtypes of PA. It is well known that APA occasionally manifests cortisol-producing capacity [1]. Previous reports indicate that roughly 10%–20% of PAs have merged mild autonomous cortisol secretion (MACS), and the patients who were diagnosed as PA associated with autonomous cortisol secretion had a high incidence of cardiovascular events [2–4]. The recent development of steroid 11b-hydroxylase (CYP11B1) and aldosterone synthase (CYP11B2) immunostaining techniques enables researchers to understand the location of cortisol- and aldosterone-producing cells in the histopathology of tumors [5]. Previous studies reported tumors with combined aldosterone- and cortisol-producing capacity on histology [6]. In contrast, two previous reports have described cases preoperatively diagnosed as bilateral PA combined with a unilateral cortisol-producing adenoma (CPA): One case had Cushing's syndrome and the other had MACS. In postoperative histopathology findings, an aldosterone-producing cell cluster (APCC)/aldosterone-producing micronodule (APM), stained by CYP11B2 antibody, was found separate from the tumor area [7–9]. In these two reports, the bilateral PAs were thought to be due to APCCs/APMs, which might be distributed in both adrenal glands without forming tumor tissue. It is described that the PA was determined to remain and drug therapy was continued after resection of CPAs in both cases.

Here, we report two cases in which a unilateral CPA and bilateral PA were diagnosed preoperatively, but after resection of the unilateral CPA, the bilateral PA unexpectedly went into remission. These cases indicate the importance of endocrinological evaluation after surgery.

## 2. Case Presentation

### 2.1. Methods

#### 2.1.1. Sample Preparation and Gene Expression Assays

Adrenal tissue was obtained from two patients who underwent adrenalectomy at Institute of Science Tokyo. The collection and storage of specimens are as described in the previous report [10]. Total RNA was extracted from the tissues using AllPrep DNA/RNA Mini (QIAGEN, Valencia, CA, USA). Total RNA was reverse transcribed into cDNA by ReverTra Ace kit (Toyobo, Osaka, Japan). The TaqMan Gene Expression Assay (Thermo Fisher Scientific, Waltham, MA, USA) was used to quantify mRNA levels of *CYP11B1* and *CYP11B2*. The TaqMan Fast Advanced Master Mix (Thermo Fisher Scientific, Waltham, MA, USA) and the QuantStudio 7 Flex Real-Time PCR System (Applied Biosystems, Thermo Fisher Scientific, Waltham, MA, USA) were used for polymerase chain reaction (PCR) amplification in a total volume of 20 µl per reaction. Each sample was assayed in duplicate and the expression of each target gene was normalized to *ACTB* as the endogenous reference gene. The primer sequence of *ACTB* was described previously [10].

### 2.2. Cases

#### 2.2.1. Case 1

**Figure 1:**
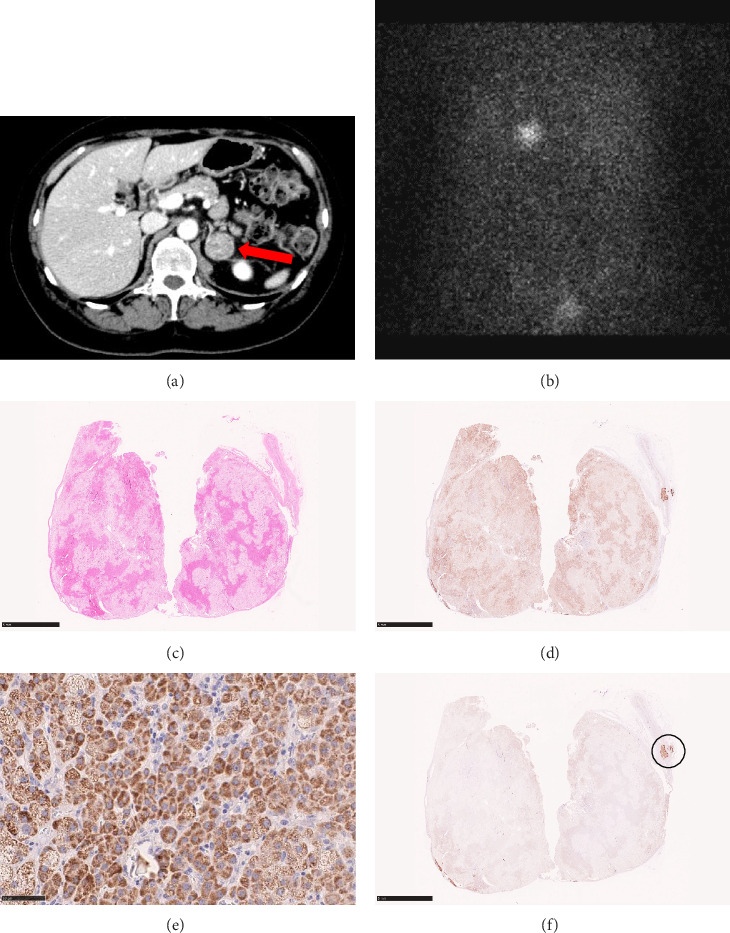
Abdominal computed tomography scan (a left adrenal tumor is indicated by a red arrow) (A) and adosterol scintigraphy of Case 1 (B). Histopathology of the adrenalectomy specimen of Case 1 by hematoxylin and eosin staining (C). Scale bar: 5 mm. Immunohistochemistry of the adrenal specimens of Case 1. Immunostaining for CYP11B1 (D, E) and CYP11B2 (F) were shown. The area circled in black consists of cells positive for CYP11B2 and is suggested to be APCC/APM. Scale bars: 5 mm (D, F), and 50 μm (E).

**Figure 2:**
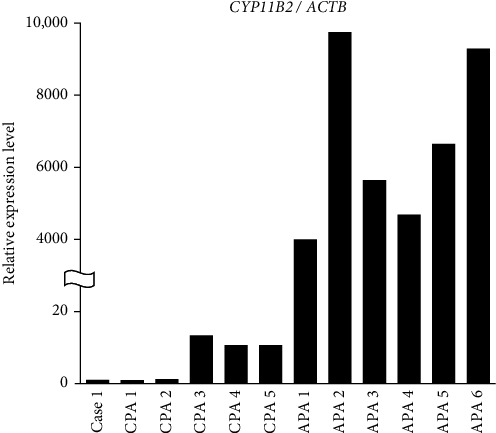
Evaluation of mRNA expression levels in six cortisol-producing adenomas (CPAs), including Cases 1 and 6 APAs. The qRT-PCR analysis of CYP11B2 was normalized to ACTB and the relative expression levels were calculated based on Case 1.

A 57-year-old woman was admitted to our hospital due to a left adrenal tumor. One year before this admission, she was diagnosed with hypertension in a medical checkup and was started on an antihypertensive medication at a nearby hospital. Abdominal ultrasonography at the hospital revealed a tumor on the left adrenal gland and she was referred to our hospital. On admission, her blood pressure and heart rate were 162/107 mmHg and 72 beats/min, respectively, with slow-release nifedipine 40 mg and doxazosin mesylate 2 mg. Her height, body weight, and body mass index were 152.2 cm, 58.6 kg, and 25.3 kg/m^2^, respectively, and she had Cushingoid features (moon face, buffalo hump, central obesity, and thin skin). The serum potassium level was 3.6 mEq/L (normal, 3.7–5.0 mEq/L). The serum cortisol level was 15.1 μg/dL (normal, 4.0–18.3 μg/dL), and the ACTH level was 2.2 pg/mL (normal, 7.2–63.3 pg/mL). The plasma renin activity (PRA) was 0.2 ng/mL/h (normal, 0.2–2.7 ng/mL/h) and plasma aldosterone concentration (PAC) was 211 pg/mL (normal, 30–159 pg/mL) with a high aldosterone renin ratio (ARR) of 1055. Abdominal computed tomography showed a left adrenal nodule measuring approximately27 mm × 25 mm (Figure 1A). Cortisol levels measured at midnight were elevated (ACTH: 1.8 pg/mL and cortisol: 15.1 μg/dL). The cortisol level was not adequately suppressed in the dexamethasone suppression test (DST;1 mg dexamethasone; cortisol 14.2 μg/dL and 8 mg dexamethasone; cortisol 15.9 μg/dL). Adosterol scintigraphy showed an accumulation in the left adrenal tumor (Figure 1B). Based on these results, we diagnosed her with Cushing's syndrome. In addition, in the furosemide upright test (FUT), PRA elevation was suppressed (1.2 ng/mL/h). In the captopril challenge test (CCT), ARR exceeded the threshold (PRA: from 0.2 to 0.1 ng/mL/h, PAC: 211–121 pg/mL, ARR: 1055–1210). The abovementioned results confirmed a diagnosis of PA and she underwent adrenal venous sampling (AVS) to differentiate the PA subtype (Table 1). The lateralized ratio (LR) remained >4 both before and after ACTH stimulation, indicating aldosterone hypersecretion from the nontumor side (right side). Concurrently, the contralateral ratio (CR) exceeded 1 in both conditions, suggesting bilateral aldosterone secretion not supporting the unilateral aldosterone secretion. Moreover, cortisol levels on the tumor side (left side) were markedly elevated (preACTH stimulation: right 11.6 μg/dL and left 121.2 μg/dL; postACTH stimulation: right 72.1 μg/dL and left 1119.7 μg/dL), implying that the aldosterone-to-cortisol ratio (PAC/F) on the tumor side was underestimated due to excessive cortisol secretion. PostACTH stimulation, PAC were comparable between the left and right sides (right: 9140 pg/mL and left: 9340 pg/mL), failing to suggest a distinct unilateral secretion pattern, finally leading to a diagnosis of bilateral forms. We finally diagnosed this patient with overt Cushing's syndrome and bilateral PA. She underwent a laparoscopic left adrenalectomy and the tumor was histologically diagnosed as an adrenocortical adenoma (Figure 1C). The tumor cells were partially positive for *CYP11B1* and negative for *CYP11B2*, which was consistent with the findings of CPA (Figure 1D-F). A *CYP11B2*-positive cell population was found in the normal adrenal cortex distant from the tumor (Figure 1F) and was suggested to be APCC/APM. Furthermore, we extracted total RNA from tumor tissue and evaluated it by quantitative reverse transcription PCR (qRT-PCR) and found that the expression level of *CYP11B2* was lower than typical APAs (*n* = 6) and comparable to typical CPAs (*n* = 5; Figure 2).

After the surgery, her blood pressure was 110–130/70–80 mmHg with slow-release nifedipine 20 mg. After 1 year of the surgery, we measured basic hormone levels and found that PRA and PAC were 1.2 ng/mL/h and 177 pg/mL, respectively, with an ARR of less than 200. In addition, we performed CCT again and her ARR was less than 200 (ARR: from 148 to 116). Based on these basic hormone levels and the loading test results, we felt that the PA was in remission.

#### 2.2.2. Case 2

**Figure 3:**
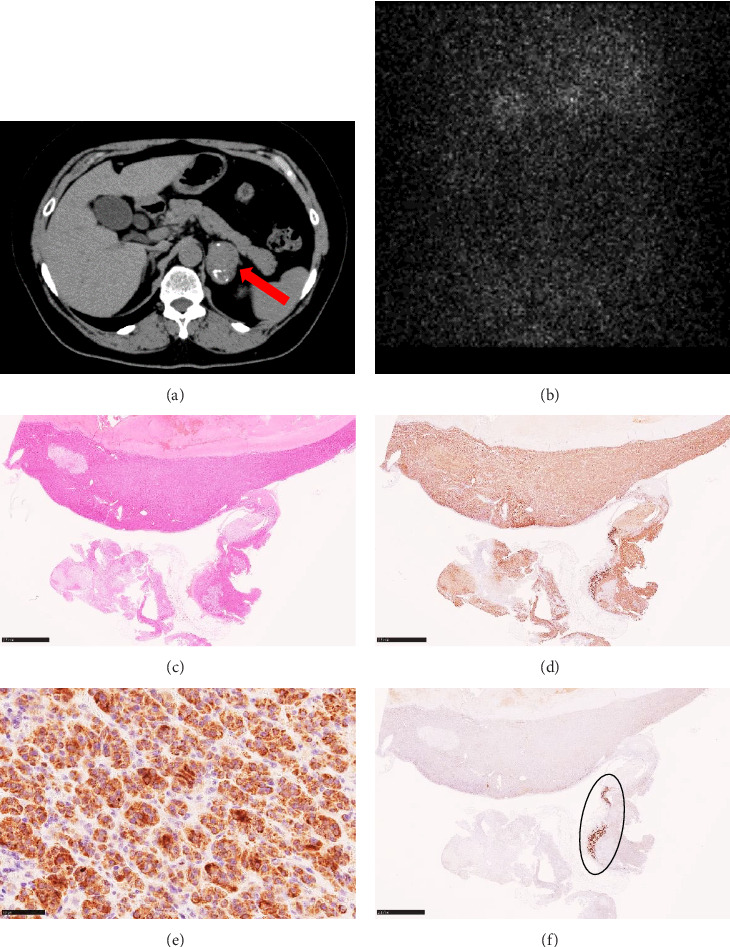
Abdominal computed tomography scan (a left adrenal tumor is indicated by a red arrow) (A) and adosterol scintigraphy of Case 2 (B). Histopathology of the adrenalectomy specimen of Case 2 by hematoxylin and eosin staining (C). Scale bar, 2.5 mm. Immunohistochemistry of the adrenal specimens of Case 2. Immunostaining for CYP11B1 (D, E), and CYP11B2 (F) were shown. The area circled in black consists of cells positive for CYP11B2 and is thought to be APCC/APM. Scale bars, 2.5 mm (D and F) and 50 μm (E).

A 67-year-old woman was admitted to our hospital due to a left adrenal tumor. She regularly visited a nearby hospital for follow-up of her gallstones and computed tomography accidentally revealed a left adrenal tumor (measuring ~38 mm **× **26 mm; Figure 3A). On admission, her height, body weight, and body mass index were 154.8 cm, 71.7 kg, and 29.9 kg/m^2^, respectively. She had no overt Cushingoid features and her blood pressure and heart rate were 148/82 mmHg and 71 beats/min without antihypertensive drugs. The serum potassium level was 4.1 mEq/L. The serum cortisol level was 13.2 μg/dL and ACTH levels was 13.2 pg/mL. PRA was 0.5 ng/mL/h, and PAC was 179 pg/mL with a high ARR of 358 Cortisol levels measured at midnight were not reduced (ACTH: 17.8 pg/mL and cortisol: 18.4 μg/dL). The cortisol level after DST was not adequately suppressed (1 mg dexamethasone: cortisol 1.9 μg/dL). Adosterol scintigraphy showed no accumulation in the left adrenal tumor (Figure 3B). According to the current guideline, the tumor satisfied the diagnostic criteria for MACS [11]. In FUT, PRA elevation was suppressed (PRA: from 0.2 to 0.6 ng/mL/h). In CCT, ARR exceeded the threshold (ARR: from 185 to 300). The abovementioned results confirmed a diagnosis of PA and she underwent AVS to differentiate the PA subtype (Table 2). The LR did not exceed four on either side, both pre- and post-ACTH stimulation, while the CR remained >1 in both conditions, indicative of bilateral forms. Additionally, postACTH stimulation cortisol levels were comparable between the left and right adrenal glands (right: 679.5 μg/dL and left: 630.8 μg/dL), suggesting that the influence of PAC/F underestimation on the tumor side was minimal. Furthermore, PAC was notably higher on the nontumor side (right: 18,800 pg/mL and left: 8,880 pg/mL), making it unlikely that the tumor side is the primary source of aldosterone excess. We diagnosed this patient with bilateral PA with a unilateral CPA. Initially, upon diagnosis, the patient expressed a reluctance towards undergoing surgery and we opted for a continued follow-up for 3 years. In these years, tumor diameter has been increasing to 47 mm ×** **36 mm, and the cortisol concentration after 1 mg DST was 2.0 μg/dL. She consented to undergo surgical intervention, and we proceeded with the laparoscopic left adrenalectomy and the tumor was histologically diagnosed as an adrenocortical adenoma (Figure 3C). The tumor cells were positive for *CYP11B1* (Figure 3D, E) and negative for *CYP11B2* (Figure 3F), consistent with the findings of a CPA. Besides, a *CYP11B2*-positive small area was found in the normal adrenal cortex distant from the tumor (Figure 3F), similar to Case 1.

Postoperatively, her blood pressure was 110–130/70–80 mmHg without antihypertensive drugs. After 1 year of surgery, we measured basic hormone levels and found that PRA and PAC were 1.5 ng/mL/h and 30.6 pg/mL, respectively, with an ARR of less than 200. Then we performed CCT again and her ARR was under 200 (ARR: from 20.4 to 4.6). Based on these basic hormone levels and the results of CCT, the PA was in remission.

## 3. Discussion

In this report, we presented two cases of bilateral PA with autonomous cortisol secretion capacity before surgery and experienced remission of PA after unilateral adrenalectomy for CPA. There have been several case reports of patients who were diagnosed with bilateral PA and Cushing's syndrome or MACS preoperatively and treated with unilateral adrenalectomy and continued drug treatment for residual PA after operation [7, 8], but there have been no reports of remission of bilateral PA after resection of the unilateral CPA. The diagnosis of bilateral PA in our cases was determined with AVS. In AVS, laterality is usually determined by the left and right aldosterone/cortisol (A/C) ratio [12]. However, in cases with autonomous cortisol secretion, as in the present cases, it is controversial. In AVS, excessive cortisol secretion lowers the A/C ratio on the tumor side, and it could affect the judgment for the laterality of aldosterone secretion [13]. The Japanese guidelines recommend comparing the adrenal vein aldosterone concentration values rather than the A/C ratio to assess the laterality [12]. In Cases 1 and 2, cortisol levels were higher and the A/C ratios were lower on the tumor side. Considering the influence of cortisol, adrenal venous PAC after ACTH loading was compared without correction with cortisol and we found that the LR ratio was <4 and not significant in both cases and even higher on the nontumor side in Case 2. Even taking into account the effect of cortisol hypersecretion, both cases did not show a laterality that would recommend unilateral adrenalectomy for PA. However, after unilateral CPA resection, PA was in remission as assessed by basal hormone levels and CCT.

We hypothesized three reasons for the unexpected remission of bilateral PA after unilateral adrenalectomy. First, both resected adrenal glands from the two cases contained small regions adjacent to the CPA stained with anti-*CYP11B2* antibody on histology. APCC, also called APM, is a cluster of cells strongly expressing *CYP11B2*, found in the adrenal zona glomerulosa, and can produce aldosterone [14]. Recent studies have shown that APCC increases with age and some have genetic mutations similar to APA [15]. Since there is a previous report that interpreted the presence of APCC in the adjacent adrenal gland of CPA as the responsible lesion for PA [8], it is speculated that regions considered to be APCC might induce autonomous aldosterone production, thereby satisfying the diagnostic criteria for PA. It is possible that the resection of the unilateral APCC may have reduced the total aldosterone secretion to the extent, where basal ARR and CCT became negative, giving the appearance of PA remission. However, it is difficult to explain why unilateral APCC, responsible for autonomous aldosterone production, did not show clear localization in AVS in both cases.

Second, the preoperative diagnosis of PA was possibly a false positive and became negative after the resection of the CPA. Hypercortisolemia can overwhelm 11*β*HSD2 activity and result in cortisol-mediated mineralocorticoid activation and subsequent intravascular volume expansion with suppression of PRA [16]. Suppression of PRA may have caused a false positive in the PA endocrine test. However, PRA suppression does not occur in all typical Cushing's syndrome cases [16], and there is a report suggesting that cortisol production in PA is rather associated with an increase in PRA [17]. In addition, especially in Case 2, diagnosed as MACS, its serum cortisol levels were not so high to suppress PRA. Considering the fact that PRA was suppressed in Case 2 despite the mild cortisol-producing capacity, one can speculate that the intermediate steroid metabolites with mineralocorticoid effect, such as deoxycorticosterone (DOC), would be produced in the resected tumor. However, reported tumors generating DOC exhibit hypertension refractory to treatment, hypokalemia, and hypoaldosteronemia [18, 19], and these manifestations were absent in Case 2.

Finally, it was possible that in both cases, the catheter insertion into the left adrenal vein (the tumor side) was inadequate. Although, in both Cases 1 and 2, the selectivity index in the left adrenal vein met the criteria of being ≥2 before ACTH stimulation and ≥5 after ACTH stimulation, indicating successful catheterization, the high cortisol secretion from the tumor side may have masked the potential failure, leading to an underestimation of aldosterone levels from tumor side. However, given that catheterization into the right adrenal vein is generally more difficult [20] and that the selectivity index in the left adrenal vein was sufficiently high, it is unlikely that the catheterization into the left adrenal vein failed in both cases. Considering that several reports have indicated that correction with metanephrines, rather than cortisol, was useful in determining laterality [21, 22], measuring metanephrines and using them to correct aldosterone levels could have been helpful in evaluating the success of AVS and in identifying laterality in our cases. The actual reason for improving bilateral PA after resection of unilateral CPA is unclear. A common feature of both cases was the absence of hypokalemia, which suggested that both cases showed a mild PA phenotype. In cases of unilateral CPA concomitant with bilateral PA, unilateral adrenalectomy may be held when the clinical symptoms of the CPA are mild, as the residual PA can persist after surgery. Williams et al. [23] demonstrated the efficacy of both unilateral and bilateral adrenalectomy in patients diagnosed with bilateral PA. In this study, at more than 12 months postoperatively, unilateral adrenalectomy achieved complete clinical remission in approximately 25% of cases and partial remission in 53%, while biochemical remission was observed in 59% and 23%, respectively. Although the underlying mechanism driving resolution following unilateral resection was not fully elucidated, a clinical phenotype of bilateral PA that could potentially benefit from unilateral adrenalectomy may exist; however, its specific characteristics remain undefined. Bilateral PA accompanied by a CPA, as observed in our cases, may be a potential candidate for surgical intervention. Therefore, the accumulation of similar cases is desirable. It is likely that in similar instances where residual PA is suspected after adrenalectomy, a reevaluation of PA should be considered.

## 4. Conclusion

After resection of a unilateral CPA from the patient with bilateral PA, the patient should be followed up with a confirmatory test to evaluate the existence of PA. Accumulating similar cases could shed light on the unknown mechanisms of this unexpected resolution of PA in our cases.

## Figures and Tables

**Table 1:** Results of adrenal venous sampling of Case 1 (tumor side: left).

	AVS hormonal values		
	Cortisol (µg/dL)	Aldosterone (pg/mL)	Aldosterone/Cortisol
Unstimulated AVS			
Inferior vena cava	7.0	73	10.43
Right adrenal vein	11.6	878	75.69
Left adrenal vein (proximal)	113.0	1940	17.17
Left adrenal vein (distal)	121.2	1720	14.19
ACTH stimulated AVS			
Inferior vena cava	20.5	118	5.76
Right adrenal vein	72.1	9140	126.77
Left adrenal vein (proximal)	1119.7	9340	8.34
Left adrenal vein (distal)	964.5	8980	9.31

*Note:* Unstimulated AVS: LR 4.40, CR 1.65. ACTH stimulated AVS: LR 13.61, CR 1.62. The higher aldosterone/cortisol value of the proximal and distal veins was adopted to calculate LR and CR.

Abbreviations: CR, contralateral ratio; LR, lateralized ratio.

**Table 2:** Results of adrenal venous sampling of Case 2 (tumor side: left).

	AVS hormonal values		
	Cortisol (µg/dL)	Aldosterone (pg/mL)	Aldosterone/Cortisol
Unstimulated AVS			
Inferior vena cava	16.1	101	6.27
Right adrenal vein	13.9	369	26.55
Left adrenal vein (proximal)	52.6	307	5.84
Left adrenal vein (distal)	43.1	399	9.26
ACTH stimulated AVS			
Inferior vena cava	29.9	263	8.80
Right adrenal vein	679.5	18,800	27.67
Left adrenal vein (proximal)	630.8	8880	14.08
Left adrenal vein (distal)	565.2	7560	13.38

*Note:* Unstimulated AVS: LR 2.86, CR 1.47. ACTH stimulated AVS: LR 1.97, CR 1.60. The higher aldosterone/cortisol value of the proximal and distal veins was adopted to calculate LR and CR.

## Data Availability

The authors declare that all supporting data are available upon reasonable request.
